# Knockdown of miR-361-5p promotes the induced activation of SHF-stem cells through FOXM1 mediated Wnt/β-catenin pathway in cashmere goats

**DOI:** 10.1080/10495398.2024.2356110

**Published:** 2024-05-28

**Authors:** Ruqing Xu, Man Bai, Yixing Fan, Yubo Zhu, Zeying Wang, Taiyu Hui, Qi Zhang, Xingwang Liu, Jialiang Zhang, Jincheng Shen, Wenlin Bai

**Affiliations:** College of Animal Science & Veterinary Medicine, Shenyang Agricultural University, Shenyang, P. R. China

**Keywords:** MiR-361-5p, FOXM1, SHF-stem cells, induced activation, cashmere goats

## Abstract

The inducing activation event of secondary hair follicle (SHF)-stem cells is considered a key biological process in the SHF regeneration, and the morphogenesis of cashmere fiber in cashmere goats. The miR-361-5p was essentially implicated in the induced activation of SHF-stem cells of cashmere goats, but its functional mechanisms are unclear. Here, we confirmed miR-361-5p was significantly downregulated in anagen SHF bugle of cashmere goats compared with that at telogen, and miR-361-5p expression was significantly lower in SHF-stem cells after activation than its counterpart before activation. Further, we found that miR-361-5p could negatively regulate the induced activation event of SHF-stem cells in cashmere goats. Mechanistically, through dual-luciferase reporter assays, miR-361-5p specifically bound with *FOXM1* mRNA in SHF-stem cells of cashmere goats and negatively regulated the expression of *FOXM1* gene. Also, through overexpression/knockdown analysis of *FOXM1* gene, our results indicated that *FOXM1* upregulated the expression of Wnt/β-catenin pathway related genes in SHF-stem cells. Moreover, based on TOP/FOP-flash Wnt report assays, the knockdown of miR-361-5p promotes the Wnt/β-catenin pathway activation through upregulating the *FOXM1* expression in SHF-stem cells. Finally, we demonstrated that miR-361-5p negatively regulated the induced activation of SHF-stem cells through *FOXM1* mediated Wnt/β-catenin pathway in cashmere goats.

## Introduction

Cashmere goats are widely distributed in the northern China. There are several excellent breeds of cashmere goats, such as Inner Mongolia cashmere goat, Liaoning cashmere goat, Tibetan cashmere goat, Hexi cashmere goat, and Shanbei white cashmere goat.^1^ As well known, cashmere goats have been reared for multi-purpose use, including cashmere, meat, skin, and wool, etc.^2^ Of them, the cashmere and meat are greater of economic significance for local farmers herdsmen.^3^ The cashmere, a kind of nature protein fiber, is derived from secondary hair follicle (SHF) of cashmere goats, and its textiles have the typical features, such as softness, comfort, shine and light, which are rather popular among consumers with widespread praise and favor.^4^^,^^5^

During the production of cashmere fibers, the induced activation of SHF-stem cells by dermal papilla cell (DPC)-derived signals was considered to play a key functional role which is closely related to the SHF regeneration, the morphogenesis of cashmere fiber with its subsequent growth.^6^^,^^7^ As matter of fact, the induced activation of SHF-stem cells is a complex process that is jointly controlled by many endogenous factors,^8^ among which, the Wnt/β-catenin signal has been identified as a key pathway.^7^^,^^9^ Also, some functional genes and molecules were revealed to participate in the induced activation process of SHF-stem cells, such as PDGFA,^10^ TNF,^11^ FOXI3,^9^ and circRNA-ZNF638.^7^ Besides, there are evidences that several miRNAs are also revealed to play roles in the induced activation of hair follicle stem cells, like miR-214,^12^ miR-339-5p,^13^ miR-29a/b1^14^ and miR-149-5p.^15^^,^^16^

The functional significance of miR-361-5p was extensively investigated in various species, and increasing evidences of lines indicated that miR-361-5p was essentially implicated in the proliferation, differentiation, and apoptosis of a variety of biological cells. For instance, it was reported that miR-361-5p promoted proliferation and inhibited apoptosis of fibroblast-like synoviocytes via targeting *ZBTB10* mRNA in rheumatoid arthritis.^17^ Also, miR-361-5p was revealed to inhibit the progress of lung cancer cell lines by targeting *FOXM1* mRNA,^18^ and regulate ovarian cancer cell proliferation and apoptosis via targeting *TRAF3* mRNA.^19^ Additionally, it was reported that miR-361-5p promoted porcine granulosa cell apoptosis through mediating *SMAD4* and *VEGFA.*^20^ In our recent investigation, we found that the miR-361-5p was also significantly implicated in induced activation event of SHF-stem cells through competitively binding with circRNA-ZNF638 and *Wnt5a* mRNAs in cashmere goats.^7^ However, the precise functional significance of miR-361-5p in the induced activation event of SHF-stem cells of cashmere goats is still unclear.

In this study, we firstly investigated the relative expression of miR-361-5p in SHF bulge region of cashmere goats, along with the expression pattern in before and after induced activation SHF-stem cells. Secondly, we evaluated the effect of miR-361-5p on the induced activation of SHF-stem cells in cashmere goats with analyzing its possible functional mechanisms. The results from this present study provided novel significant information for revealing the molecular mechanisms on the induced activation event of SHF-stem cells in cashmere goats. Also, our results provided molecular basis for artificially promoting SHF regeneration, the morphogenesis and growth of cashmere fiber to improve the cashmere yield and quality in cashmere goats.

## Materials and methods

### Total RNA of SHF bugle and cell non-contacting co-culture assay

All experiment procedures were approved by Experimental Animal Ethics and Welfare Committee of Shenyang Agricultural University (approval number: 201606005) and the all experiments were performed following the approved procedures. The total RNA of SHF bugle of cashmere goat was used that was isolated in our another investigation.^7^ The SHF-stem cells of cashmere goats were co-cultured with DPCs of cashmere goats for induced activation in an established Transwell co-culture system as described in previous study,^8^ where the SHF-stem cells (passage 3) were seeded in the lower chamber while the DPCs (passage 3) were seeded in the upper chamber. The used SHF-stem cells and DPCs of cashmere goats were stored in our laboratory. The non-contacting co-culture of SHF-stem cells and DPCs was carried out in fresh DMEM/F12 medium (Hyclone, Logan, UT, USA) supplemented with fetal bovine serum (10%), which is under a humidified atmosphere at 37 °C and 5% CO_2_. The culture media was replaced every 48 h.

### Overexpression and knockdown of miR-361-5p and *FOXM1* mRNA

The mimics/inhibitor of miR-361-5p with their negative control (NC) were purchased from Shanghai GenePharma Co., Ltd (Shanghai, China). For generating the overexpression vectors of *FOXM1* gene, the cDNA of cashmere goat SHFs was synthesized by PrimeScript™ 1st Strand cDNA Synthesis Kit (TaKaRa, Dalian, China). For amplifying the CDS sequence of *FOXM1* mRNA, the primers were designed based on the goat *FOXM1* mRNA sequence (GenBank accession no. NM_001314325.1). Subsequently, the PCR amplifications were carried out to obtain the CDS sequence of goat FOXM1 gene using the Phanta Max Super-Fidelity DNA Polymerase (Vazyme, Nanjing, China). The obtained PCR products were cloned into the pcDNA3.1(+) vectors. The small interference RNA targeting *FOXM1* mRNA (siRNA-FOXM1) and its negative control (siRNA-NC) were commercially designed and synthesized by Shanghai GenePharma. Co., Ltd (Shanghai, China).

### Dual-luciferase reporter assays

For confirming the specific binding of miR-361-5p with *FOXM1* mRNA in SHF-stem cells, we amplified the predicted targeted fragments (328-bp) of *FOXM1* mRNA (FOXM1-WT) of cashmere goat which contained the predicted binding site of miR-361-5p. The *FOXM1* mutants (FOXM1-MUT) were generated using the Fast Site-Directed Mutagenesis Kit (Tiangen, Beijing, China), within which the predicted binding sites of miR-361-5p seed region were mutated with their corresponding complementary bases. The obtained products of *FOXM1* mRNA targeted fragments (or FOXM1-MUT) were cloned into the pmirGLO vector (Promega, Madison, WI, USA). The miR-361-5p mimics or its negative control (miRNA NC) and pmirGLO-FOXM1-WT/pmirGLO-FOXM1-MUT were co-transfected into SHF-stem cells (passage 3) using the Lipofectamine 2000 (Invitrogen, Carlsbad, CA, USA). The co-transfected cells were subjected to non-contacting co-culture as described above. After 48 h, the cells were harvested followed by a detecting of the luciferase activities using dualluciferase reporter system (Promega, Madison, WI, USA). To estimated the relative luciferase activity, the firefly luciferase activity was normalized by the renilla luciferase activity to eliminate the potential bias in transfection efficiency differences among the analyzed cells.

### Quantitative real-time PCR (qPCR)

The total RNA was isolated from SHF bulge or cell samples using the RNAiso reagent kit (TaKaRa, Dalian, China) following the manufacturer’s recommendation. For miR-361-5p expression detection, the first strand cDNAs was synthesized using the OneStep PrimeScript microRNA cDNA synthesis kit (TaKaRa, Dalian, China). The sense primer of miR-361-5p was designed based on its mature sequence and the anti-sense primer of miR-361-5p (being universal to all miRNAs) was obtained along with the kits (TaKaRa, Dalian, China). The *U6* (internal reference gene) primer pair was designed in a previous publication.^21^ For the expression detection of mRNAs of related genes, the first strand cDNA was synthesized by the M-MuLV cDNA synthesis kit (Sangon, Shanghai, China) with random primers. The GAPDH gene was used as the internal control. The primers were designed using the Primer Premier 5.0 procedure (http://www.premierbiosoft.com). In this study, all used primers are listed in Table 1. The qPCR assay was performed in a light Cycler 480 realtime PCR system (Roche Diagnostics, Germany). In a 25 μL final volume, the qPCR amplication was carried out using TB Green Premix Ex Taq II (Tli RNaseH Plus; TaKaRa, Dalian, China). The reaction assay was composed of first-strand cDNA solution (2.0 μL), TB Green Premix Ex Taq II (12.5 μL), each primer (10 μM, 1.0 μL), and PCR-grade ddH_2_O water (8.5 μL). All reactions were performed at least in three replicates. The relative expression of mRNA or miRNA was calculated with the 2*^–ΔΔCt^*method described by Livak and Schmittgen.^24^

**Table 1.** Detail information of PCR primers used in the study with the corresponding amplicon size and annealing temperature.

Gene/miRNA name	Reference	Sequence (5′–3′)^a^	Primer length (nt)	Amplicon size (bp)	Annealing temperature (°C)
miR-361-5p	MIMAT0036171 in miRNAsong^b^	F: CGTTATCAGAATCTCCAGGGGTAC	24	Not available	60
*U6*	Han et al., 2020^21^	F: CGCTTCGGCAGCACATATAC, R:AAATATGGAACGCTTCACGA	20 20	Not available	55
*CK6*	Yin et al., 202^2^ ^7^	F:AGTTTGCCTCCTTCATCG, R:GGTTCTGCTTCACGGTCTT	18 19	111	53
*Ki67*	Yin et al., 2023^7^	F:AGGAAGTAGCCAGACTGAGGG, R:GCATCGTGGTTTGCTGTGAA	21 20	143	56
*Sox9*	Yin et al., 2023^7^	F:GGTGCTCAAGGGCTACGACTGG, R:GCGTTGTGCAGGTGCGGGTA	22 20	162	60
*CD34*	Yin et al., 2023^7^	F:GAAGATGTCAGCAGCCACCAG, R:GGCGGTTCATCAGGAAATAGCAC	21 23	112	56
*CD200*	Yin et al., 2023^7^	F:TTGGAAGATGAGGCGTGTTA, R:AGCATTGGCAGAGCAAGTGA	20 20	156	54
*GAPDH*	Yin et al., 2023^7^	F:TGAACCACGAGAAGTATAACAACA, R:GGTCATAAGTCCCTCCACGAT	24 21	125	53
*FOXM1* (for qPCR)	NM_001314325.1 in GenBank^c^	F:CAAGAGGTGGAAGAAAAGGAGA, R:ATCAGGGCCATGTAGGAGTATG	22 22	140	56
*FOXM1* (for CDS binding with miR-361-5p)	NM_001314325.1 in GenBank^c^	F:AGTGCCACCCCACTGAAA R:AGGAGCCCTGCCTCTACC	18 18	328	58
*CTNNB1*	XM_018066894.1 in GenBank	F:GTATGAGTGGGAGCAGGGGTTT, R:TGGACGTTAGTGGGATGAGCAG	22 22	180	57
*LEF1*	Zhu et al., 2021^23^	F:CCACCTCTTGGCTGGTTTTC, R:TTTGGCTCCTGCTCCTTTCT	20 20	176	56
*c-Myc*	Z68501.1 in GenBank	F:TAAGTTGGACAGTGGCAGGG, R:GGTCACGAAGAGCAAAAAAG	20 20	164	54
*CCND1*	XM_018043271.1 in GenBank	F:GCCCTCGGTGTCCTACTT, R:CTCCTCCTCGCACTTCTG	18 18	112	54
*SFRP2*	XM_013970497.2 in GenBank	F:TCACATAAAGGAAAAACCCACC, R:CAAACCAGAAAAATCAAGCCAG	22 22	146	55

^a^F: forward, R: reverse.

^b^miRNAsong:https://www.med.muni.cz/histology/miRNAsong.

^c^GenBank: https://www.ncbi.nlm.nih.gov

### TOP/FOP-flash Wnt report assays

The β-catenin-driven transcription was assessed using the TOP/FOP-flash plasmids (MiaoLing, Wuhan, China) and the mimics/inhibitor of miR-361-5p where the FOP flash (being TOP-flash mutant) reporter plasmids were used as negative control. The Renilla luciferase reporter plasmids (Beyotime, Shanghai, China) were transiently co-transfected into SHF-stem cells using the Lipofectamine 2000 (Invitrogen, Carlsbad, CA, USA). Following transfection for 48 h, the activities of firefly and Renilla luciferase reporters were measured using a Dual Luciferase Assay Kit (Promega, Madison, WI, USA) according to the manufacturer’s recommendations.

Ultimately, the TOP-Flash reporter activity is presented as the firefly luciferase activity being normalized to Renilla luciferase activity.

### Data statistical analysis

The obtained data of related gene expression and luciferase activity was analyzed using the SPSS 17.0 procedure (SPSS Inc., Chicago, IL, USA). The graphical representations were carried out using the GraphPad Prism 8 software (GraphPad Software Inc., San Diego CA, USA). The difference between two groups was compared using the unpaired Student’s *t*-test. ‘*’, ‘**’ and ‘***’ stand for the obtained *p* value less than 0.05, 0.01 and 0.001, respectively.

## Results and discussion

### Expression changes of miR-361-5p in SHF bulge of cashmere goats and its functional significance in the induced activation of SHF-stem cells

**Figure 1.**
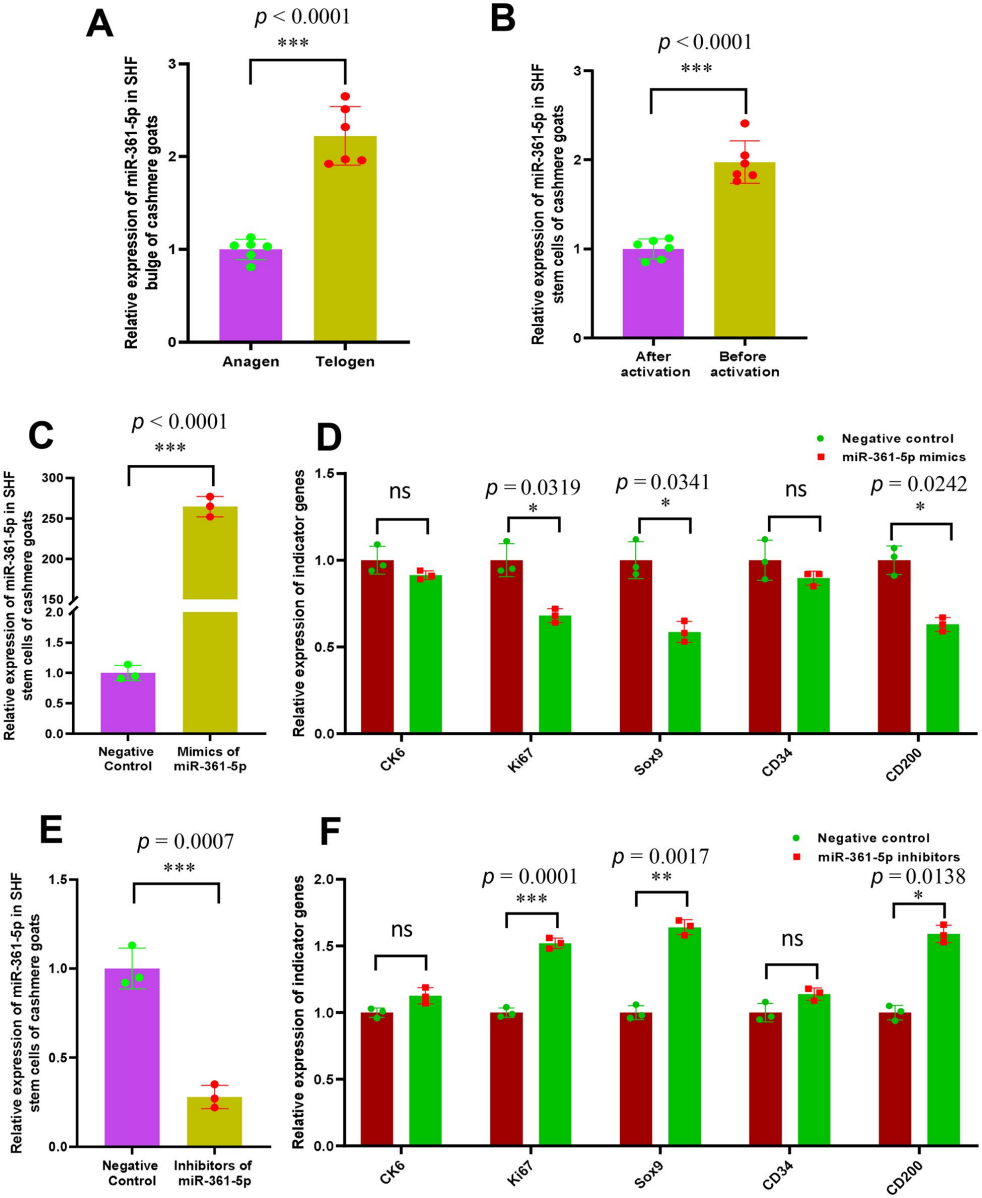
The relative expression of miR-361-5p in the SHF bugle and stem cells before and after activation with its functional significance in the induced activation of SHF-stem cells of cashmere goats. (A) Relative expression of miR-361-5p in cashmere goat SHF bugle at anagen and telogen stages. (B) Relative expression of miR-361-5p before and after induced activation of SHF-stem cells. (C) Overexpression efficiency analysis of miR-361-5p in SHF-stem cells of cashmere goats. (D) Overexpression of miR-361-5p led to the significant decreasing expression of three indictor genes in SHF-stem cells including Ki67, Sox9 and CD200. (E) Konckdown efficiency analysis of miR-361-5p in SHF-stem cells of cashmere goats. (F) Konckdown of miR-361-5p led to the significant increasing expression of three indictor genes in SHF-stem cells including Ki67, Sox9 and CD200. The ‘*’, ‘**’ and ‘***’ stand for indicating significant difference with *p* < 0.05, *p* < 0.01 and *p* < 0.001, respectively.

The relative expression of miR-361-5p in SHF bugle of cashmere goats was detected at both anagen and telogen stages. We found that miR-361-5p was significantly down-expressed in anagen SHF bugle of cashmere goats compared with its counterpart at telogen (Fig. 1A). Also, it was recorded that miR-361-5p expression was significantly lower in SHF-stem cells after activation than its counterpart before activation (Fig. 1B). As well known, unlike telogen, the anagen SHF-stem cells of bulge region are under boom status of induced activation by the DPC-derived signals which is essentially important in driving the SHF regeneration along with the cashmere fiber morphogenesis and its growth.^6^^,^^7^ Therefore, we assume that miR-361-5p may negatively regulate the induced activation event of SHF-stem cells in cashmere goats.

To define this assumption, the SHF-stem cells of cashmere goats were transfected with miR-361-5p mimics and its negative control. As expected, the miR-361-5p level after its over-expression was significantly higher than that in the negative group (Fig. 1C). Interestingly, the overexpression of miR-361-5p led to significant decrease in expression of several tested indicator genes (on induced activation of SHF-stem cells) in SHF-stem cells including *Ki67*, *Sox9* and *CD200* (Fig. 1D). Meanwhile, we also performed a knockdown analysis of miR-361-5p in SHF-stem cells via transfecting its inhibitors, and its significant knockdown was verified by qPCR analysis (Fig. 1E). As shown in Fig. 1F, the knockdown of miR-361-5p led to a significant increase in expression of three indicator genes: *Ki67*, *Sox9* and *CD200*. Moreover, it was recorded that Ki67 had been demonstrated to label bulge hair follicle stem cells during early phases of activation when hair follicle stem cells under slow-cycling status begin to divide and differentiate.^25^ The Sox9 expression was detected mainly in the outer root sheath and the bulge region of hair follicle, and it was reported that Sox9 play a key role in hair differentiation.^25^ Also, the CD200 expression is mainly concentrated in keratinocytes that comprises the outer root sheath of hair follicles.^26^ Taken together with our results from both miR-361-5p overexpression (Fig. 1D) and its knockdown (Fig. 1F) in SHF-stem cells, it can be inferred that miR-361-5p may negatively regulate the induced activation process of SHF-stem cells in cashmere goats through certain mechanisms.

### miR-361-5p directly binds with *FOXM1* mRNA and downregulates its expression in SHF-stem cells

**Figure 2.**
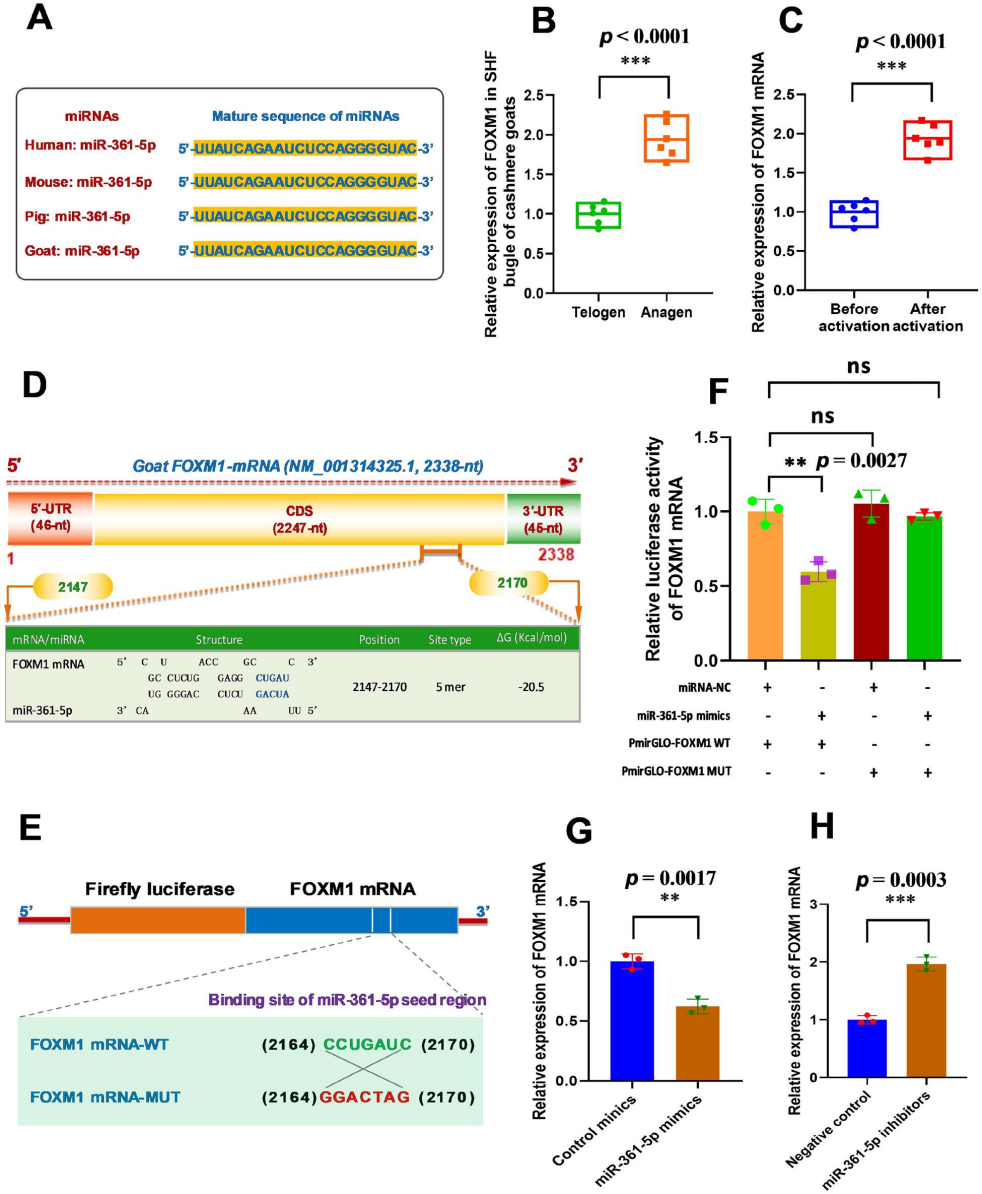
MiR-361-5p directly binds with FOXM1 mRNA and negatively regulates its expression in SHF-stem cells of cashmere goats. (A) Conservative analysis of miR-361-5p mature sequences among mammal species. (B) Relative expression of *FOXM1* mRNA in cashmere goat SHF bugle at anagen and telogen stages. (C) Relative expression of *FOXM1* mRNA before and after induced activation of SHF-stem cells. (D) A diagram of goat *FOXM1* mRNA with the prediction of binding sites of miR-361-5p in the *FOXM1* mRNA. The nucleotide positions are indicated based on the goat *FOXM1* mRNA sequence with accession number XM_001314325.1 at NCBI online database (https://www.ncbi.nlm.nih.gov). (E) The construction strategies of *FOXM1* mRNA mutant (*FOXM1* mRNA-MUT) for the miR-361-5p binding sites. (F) MiR-361-5p directly binds with *FOXM1* mRNA. (G) Overexpression of miR-361-5p led to the significant decreasing expression of *FOXM1* mRNA in SHF-stem cells. (H) Knockdown of miR-361-5p led to the significant increasing expression of *FOXM1* mRNA in SHF-stem cells. The ‘**’ and ‘***’ stand for indicating significant difference with *p* < 0.01 and *p* < 0.001, respectively.

It is widely accepted that miRNAs can directly binds with the mRNA of their target genes usually driven through a sequence-based complementary base pairing model, which leads to an inhibiting of their target mRNAs at the post-transcriptional level. In several investigations on cancer cells, it was confirmed that miR-361-5p directly binds with *FOXM1* mRNA and inhibits its expression.^18^^,^^27^^,^^28^ Furthermore, miR-361-5p was revealed to be highly conservative among species (Fig. 2A). This promotes us to ask whether miR-361-5p also directly binds with *FOXM1* mRNA in SHF-stem cells of cashmere goats. We firstly tested the expression pattern of *FOXM1* in SHF bugle of cashmere goats, as well as, SHF-stem cells before and after activation. The obtained results indicated that *FOXM1* was significantly upregulated in anagen SHF bugle of cashmere goats compared with its counterpart at telogen (Fig. 2B). Moreover, *FOXM1* expression was significantly higher in SHF-stem cells after activation than its counterpart before activation (Fig. 1C). Taken together with the expression pattern of miR-361-5p in SHF bugle of cashmere goats (Fig. 1A), and SHF-stem cells before and after activation (Fig. 1B), there seems to be a negative expression relationship between miR-361-5p and *FOXM1* mRNA.

Thus, we performed a screening for potential binding sites of miR-361-5p on goat *FOXM1* mRNA through bioinformatics analysis. As presented in Fig. 2D, a potential binding site of miR-361-5p was revealed on the *FOXM1* mRNA with a 5 mer binding type in its seed region. To further confirm this specific interaction of between miR-361-5p and *FOXM1* mRNA, we conducted a dualluciferase reporter assay in SHF-stem cells of cashmere goats. We constructed luciferase reporter vector of *FOXM1* mRNA containing the predicted binding site of miR-361-5p (PmirGLO-FOXM1-WT). Also, a corresponding mutant luciferase report vector of *FOXM1* mRNA (PmirGLO-FOXM1-MUT) was constructed which contained an antisense mismatch base of 7-nt in seed binding region of miR-361-5p (Fig. 2E). As shown in Fig. 2F, the relative luciferase activity was found to decrease when PmirGLO-FOXM1-WT and miR-361-5p mimics were co-transfected, but not in the co-transfecting group of PmirGLO-FOXM1-MUT and miR-361-5p (Fig. 2F). Moreover, the overexpression of miR-361-5p through its mimic transfection caused the decrease of the *FOXM1* mRNA expression in SHF-stem cells (Fig. 2G), whereas the knockdown of miR-361-5p through its inhibitor transfection was found to lead to a significant increase of *FOXM1* mRNA expression in SHF-stem cells (Fig. 2H). These results demonstrated that miR-361-5p specifically bound with *FOXM1* mRNA in SHF-stem cells of cashmere goats, and negatively regulated the expression of *FOXM1* gene. More recently, it was also reported that miR-361-5p directly bound with *Wnt5a* mRNA thereby regulating the induced activation of SHF-stem cells in cashmere goats.^7^ This is not surprising as a single miRNA theoretically may bind with over one hundred mRNA targets.^29^^,^^30^ This means that miR-361-5p and their potential mRNA targets may represent remarkably diverse regulatory networks in SHF-stem cells of cashmere goats as described by Mardaryev and colleagues.^31^ Thus, we suggest that other potential mRNA targets of miR-361-5p should be further investigated in SHF-stem cells, which may have essentially significance for fully revealing the molecular mechanisms on the induced activation of SHF-stem cells in cashmere goats.

In addition, here, it is worth noting that the binding site of miR-361-5p within *FOXM1* mRNA is located in the coding sequence (CDS) region close to its 3′-UTR region (Fig. 2D), which seems to go against the widely accepted viewpoint that a miRNA generally binds to the 3′-UTR of its target mRNAs.^32^ However, the binding sites of miRNAs have also been reported in the target gene 5′-UTR^33^ and CDS region.^34^ Our results also further supported the previous findings that miRNAs can bind to a variety of sites of their target mRNAs through both canonical and noncanonical base pairing rules.^35^

### FOXM1 upregulates the expression of wnt/β-catenin pathway related genes in SHF-stem cells

**Figure 3.**
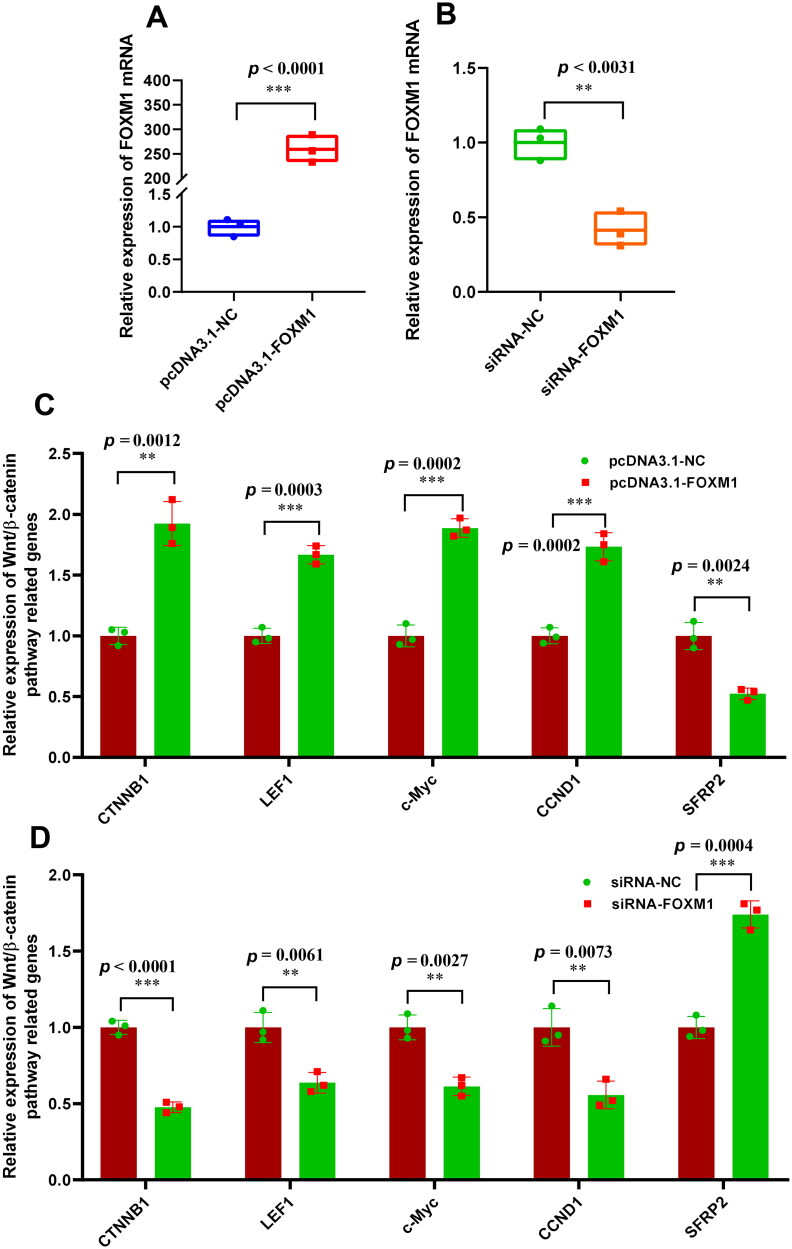
Effects of FOXM1 on the expression of wnt/β-catenin pathway related genes in SHF-stem cells. (A) Overexpression efficiency analysis of *FOXM1* in SHF-stem cells of cashmere goats. (B) Konckdown efficiency analysis of *FOXM1* in SHF-stem cells of cashmere goats. (C) Overexpression of *FOXM1* led to the significant increasing expression of wnt/β-catenin pathway related genes in SHF-stem cells including *CTNNB1*, *LEF1*, *c-Myc*, and *CCND1*. (D) Knockdown of FOXM1 led to the significant decreasing expression of Wnt/β-catenin pathway related genes in SHF-stem cells including *CTNNB1*, *LEF1*, *c-Myc*, and *CCND1.* The ‘**’ and ‘***’ stand for indicating significant difference with *p* < 0.01 and *p* < 0.001, respectively.

It is well known that the activation of Wnt/β-catenin pathway plays a pivotal role in promoting the induced activation of SHF-stem cells.^7^^,^^9^ Whereas the *FOXM1* is thought to be a key switch for activating Wnt/β-catenin pathway.^36^^,^^37^ In this study, as we indicated above that the knockdown of miR-361-5p promotes the induced activation of SHF-stem cells in cashmere goats (Fig. 1F), moreover, miR-361-5p directly binds with *FOXM1* mRNA and inhibits its expression (Fig. 2G). This promotes us ask whether the observed negative effect of miR-361-5p on the induced activation process of SHF-stem cells in cashmere goats (Fig. 1D and 1F) may be achieved through FOXM1 mediated Wnt/β-catenin pathway. Thus, we firstly performed gain- and loss-of-function analysis of *FOXM1* in SHF-stem cells of cashmere goats. The significant overexpression (Fig. 3A) and knockdown of *FOXM1* (Fig. 3B) were verified in SHF-stem cells of cashmere goats through the use of pcDNA 3.1 vector and siRNA-*FOXM1*, respectively. Further, our results indicated that the overexpression of *FOXM1* significantly upregulated the mRNA level of *CTNNB1*, *LEF1*, *c-Myc*, *CCND1*, and downregulated the *SFRP2* mRNA level in SHF-stem cells (Fig. 3C). Conversely, the knockdown of *FOXM1* significantly downregulated the mRNA level of *CTNNB1*, *LEF1*, *c-Myc*, *CCND1*, and upregulated the SFRP2 mRNA level in SHF-stem cells (Fig. 3D).

FOXM1, a member of the Forkhead box transcription factor family, is widely expressed in embryonic tissues,^22^ and is essentially implicated in the self-renewal and proliferation of various stem cells, such as neural stem cells^38^ and Glioma Stem Cells.^39^ Although the functional roles of FOXM1 in SHF-stem cells of cashmere goats remains unclear, it has been reported that FOXM1 promotes the activation of Wnt/β‐catenin pathway in a rat renal tubular epithelial cells,^36^ which is highly similar to our observed results in SHF-stem cells of cashmere goats (Fig. 3C and 3D). Interestingly, the Wnt/β-catenin signaling was revealed to serve as the most key pathway in the induced activation of SHF-stem cells.^7^^,^^9^^,^^40^ Taken together with our results, it can be suggested that FOXM1, as a positive regulator of Wnt/β-catenin signaling pathway, plays essential roles in regulating the induced activation of SHF-stem cells of cashmere goats.

### Knockdown of miR-361-5p promotes the wnt/β-catenin pathway activation through upregulating the FOXM1 expression in SHF-stem cells

**Figure 4.**
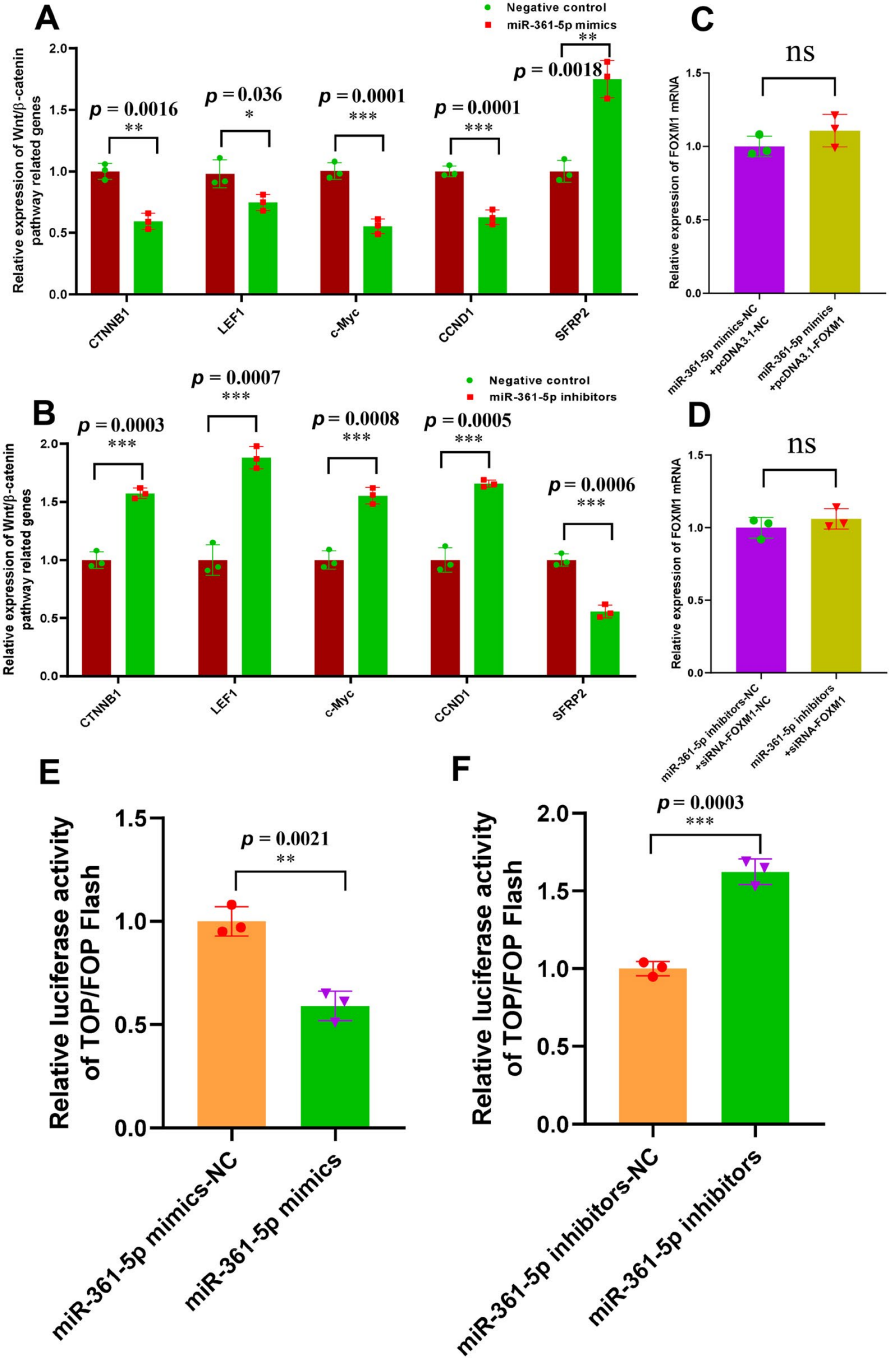
MiR-361-5p negatively regulates the wnt/β-catenin pathway activation through FOXM1 in SHF-stem cells. (A) Overexpression of miR-361-5p led to the significant decreasing expression of wnt/β-catenin pathway related genes in SHF-stem cells including CTNNB1, LEF1, c-Myc, and CCND1. (B) Knockdown of miR-361-5p led to the significant increasing expression of wnt/β-catenin pathway related genes in SHF-stem cells including *CTNNB1*, *LEF1*, *c-Myc*, and *CCND1*. (C) The overexpression of *FOXM1* rescued its mRNA expression level when miR-361-5p mimics was co-transfected in the SHF-stem cells. (D) The knockdown of FOXM1 balanced the up-regulation of *FOXM1* mRNA when miR-361-5p inhibitor was co-transfected in SHF-stem cells. (E) The overexpression of miR-361-5p significantly downregulated the activity of β-catenin-driven transcription in the SHF-stem cells with the TOP/FOP flash assay. (F) The knockdown of miR-361-5p significantly upregulated the activity of β-catenin-driven transcription in the SHF-stem cells with the TOP/FOP flash assay. The ‘*’, ‘**’ and ‘***’ stand for indicating significant difference with *p* < 0.05, *p* < 0.01 and *p* < 0.001, respectively.

Based on our above findings that miR-361-5p directly bound with *FOXM1* mRNA (Fig. 2E and 2F), and downregulated its expression in SHF-stem cells of cashmere goats (Fig. 2G and 2H). Whereas, FOXM1 positively regulated the activation of Wnt/β‐catenin pathway in SHF-stem cells of cashmere goats, which was verified through assessing the expression changes of the Wnt/β-catenin pathway related genes in SHF-stem cells (Fig. 3C and 3D). These results above from this study drove us ask whether that miR-361-5p negatively regulates the activation of Wnt/β-catenin pathway through downregulating the *FOXM1* expression in SHF-stem cells of cashmere goats. To define this assumption, we further conducted gain- and loss-of-function analysis of miR-361-5p in SHF-stem cells of cashmere goats with the expression measure of Wnt/β-catenin pathway related genes. As shown in Fig. 4A, the overexpression of miR-361-5p significantly downregulated the mRNA level of *CTNNB1*, *LEF1, c-Myc*, *CCND1*, and upregulated the *SFRP2* mRNA level in SHF-stem cell (Fig. 4A). On the contrary, the knowdown of miR-361-5p significantly upregulated the mRNA level of *CTNNB1*, *LEF1*, *c-Myc*, *CCND1*, and downregulated the *SFRP2* mRNA level in SHF-stem cells (Fig. 4B). Meanwhile, we found that the overexpression of *FOXM1* rescued the its mRNA expression level when miR361-5p mimics was co-transfected in the SHF-stem cells (Fig. 4C), and the knockdown of *FOXM1* balanced the up-regulation of *FOXM1* mRNA when miR-361-5p inhibitor was co-transfected in SHF-stem cells (Fig. 4D).

**Figure 5.**
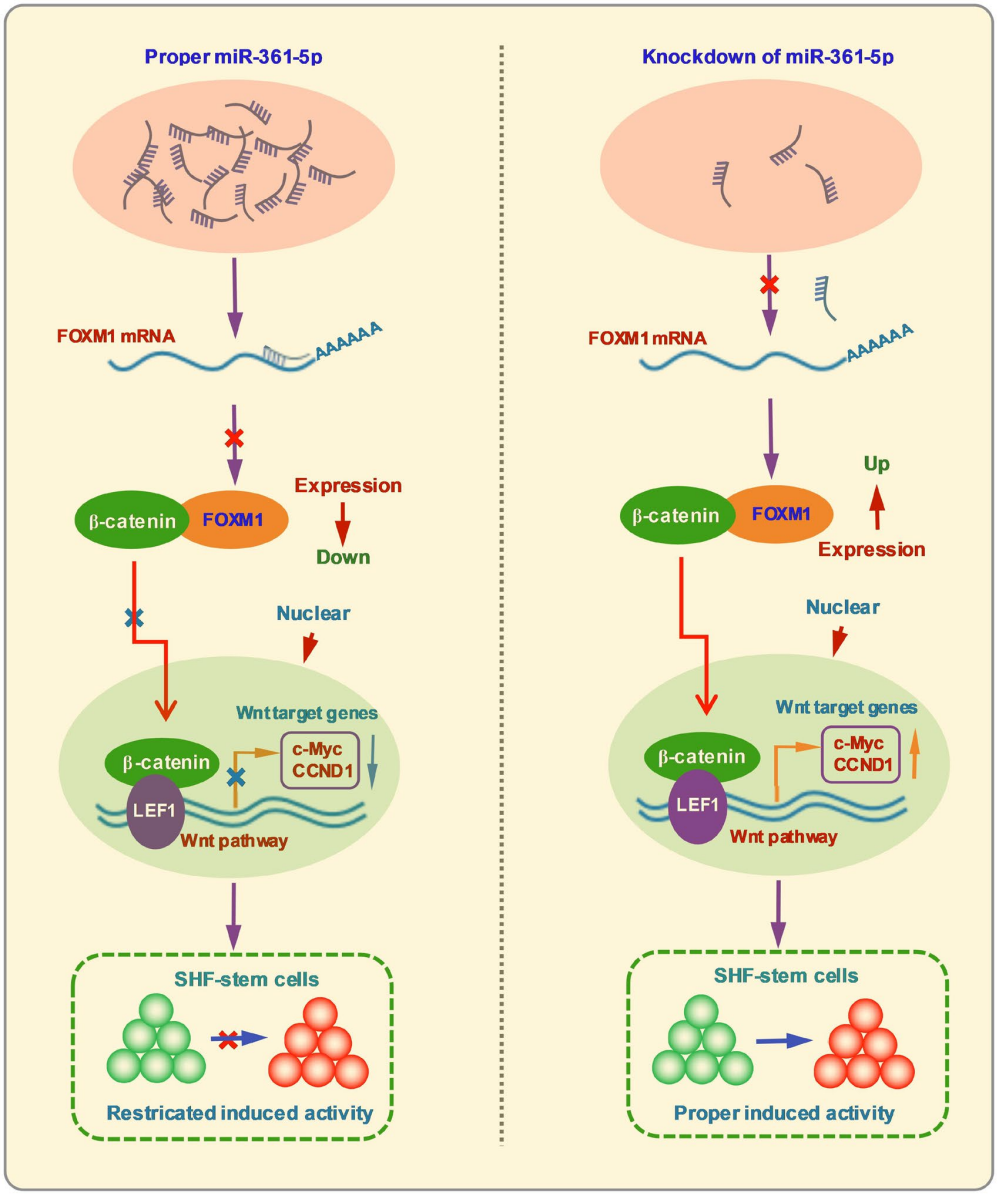
A summary schematic diagram of functional role of miR-361-5p in negatively regulating the induced activation of SHF-stem cells in cashmere goats through FOXM1 mediated wnt/β-catenin pathway.

Further, we analyzed the luciferase activity of TOP/FOP flash in SHF-stem cells. As a result, the activity of β-catenin-driven transcription was revealed to significantly decrease through transfecting the miR-361-5p mimics in SHF-stem cells (Fig. 4E), whereas the activity of β-catenin-driven transcription was found to significantly increase by transfecting the miR-361-5p inhibitors in SHF-stem cells (Fig. 4F). Thus, it can be suggested that miR-361-5p could play a negatively regulatory role in the Wnt/β-catenin signaling pathway. Previously, it was reported that FOXM1 is a downstream component of Wnt signaling and can bind directly with β-catenin, thereby enhancing the nuclear localization of β-catenin and its transcriptional activity.^41^ Here, we showed that *FOXM1* mRNA was a direct target of miR-361-5p (Fig. 2E and 2F), and its expression was upregulated in SHF-stem cells by the knockdown of miR-361-5p (Fig. 2H). Meanwhile, the knockdown of miR-361-5p could upregulate the expression level of Wnt/β-catenin pathway related genes including *CTNNB1*, *LEF1*, *c-Myc*, and *CCND1* in SHF-stem cells (Fig. 4B). Although it is not yet known whether miR-361-5p is directly implicated in Wnt/β-catenin pathway, our results implied that miR-361-5p negatively regulated the Wnt/β-catenin pathway by targeting FOXM1 in SHF-stem cells. Thus, taken together with above results, it can be inferred that knockdown of miR-361-5p promotes the induced activation of SHF-stem cells through FOXM1 mediated Wnt/β-catenin pathway in cashmere goats (Fig. 5). In the present study, however, it is worth pointing out: 1) the investigation was performed in SHF-stem cells of cashmere goats *in vitro*; 2) regarding the expression of *FOXM1* and Wnt/β-catenin pathway related genes, we merely detected the mRNA level, but not at protein level. Thus, the observed functional role of miR-361-5p above should be further verified in SHF-stem cells of cashmere goats *in vivo* with the corresponding expression detection of the implicated genes at protein level.

## Conclusions

Here, we confirmed that miR-361-5p was significantly down-expressed in anagen SHF bugle of cashmere goats compared with that at telogen. Further, we showed that the knockdown of miR-361-5p could contribute the proper induced activation of SHF-stem cells in cashmere goats, which can be achieved via FOXM1 mediated Wnt/β-catenin signaling pathway.
